# Structural study and Hirshfeld surface analysis of (*Z*)-4-(2-meth­oxy­benzyl­idene)-3-phenyl­isoxazol-5(4*H*)-one

**DOI:** 10.1107/S2056989021004308

**Published:** 2021-04-27

**Authors:** Assia Benouatas, Rima Laroum, Noudjoud Hamdouni, Wissame Zemamouche, Abdelmadjid Debache, Ali Boudjada

**Affiliations:** a Laboratoire de Cristallographie, Département de Physique, Université Mentouri-Constantine, 25000 Constantine, Algeria; b Laboratoire de Synthèse de Molécules, d’Intérêts Biologiques, Département de Chimie, Université Mentouri-Constantine, 25000 Constantine, Algeria

**Keywords:** crystal structure, meth­oxy­benzyl­idene, isoxazole, hydrogen bonding, π–π stacking, Hirshfeld surface analysis

## Abstract

The title compound, C_17_H_13_NO_3_, adopts a *Z* configuration about the C=C bond. The isoxazole and meth­oxy­benzyl­idene rings are almost coplanar with a dihedral angle of 9.63 (7)° between them. In contrast, the phenyl substituent is twisted significantly out of the plane of the oxazole ring, with the two rings inclined to each other by 46.22 (4)°. The crystal structure features C—H⋯O, C—H⋯N and C—H⋯π hydrogen bonds and π–π contacts. An analysis of the Hirshfeld surfaces points to the importance of H⋯H, H⋯C/C⋯H and H⋯O/O⋯H contacts. The included surface areas of the title compound were compared to those of the isomeric structure (*Z*)-4-(4-meth­oxy­benzyl­idene)-3-phenyl­isoxazol-5(4*H*)-one [Zhang *et al.* (2015 ▸). *CrystEngComm*, **17**, 7316–7322].

## Abstract 2

The asymmetric unit of the title compound contains one mol­ecule and the mol­ecule adopts a *Z* configuration about the C=C bond. The crystal structure features C—H⋯O and C—H⋯N hydrogen bonds together with C—H⋯π contacts and π–π stacking inter­actions. The crystal packing was further investigated by Hirshfeld surface analysis and the included surface areas from the title compound and an isomeric form were also investigated.

## Chemical context   

Isoxazolones are known to be inhibitors of the factorization of tumor necrosis alpha (TNF-α) (Laughlin *et al.*, 2005 ▸), anti­microbial agents (Mazimba *et al.*, 2014 ▸), as drugs for the treatment of cerebrovascular disorders and as muscle relaxants. In agriculture, they are used as herbicides (Guo, *et al.*, 2020 ▸) and fungicides (Miyake *et al.*, 2012 ▸). They undergo various chemical transformations (Batra *et al.*, 1994 ▸) and are excellent inter­mediates in the synthesis of various heterocycles, including pyrido­pyrimidines (Tu *et al.*, 2006 ▸), quinolines (Abbiati *et al.*, 2003 ▸) and polycycles (Badrey & Gomha, 2014 ▸). Because of their importance, these compounds have been studied extensively and several procedures for their synthesis are described using a three-component polycondensation between an aromatic aldehyde, ethyl aceto­acetate and hydrox­ylamine hydro­chloride under different conditions (Liu *et al.*, 2011 ▸; Fozooni *et al.*, 2013 ▸).
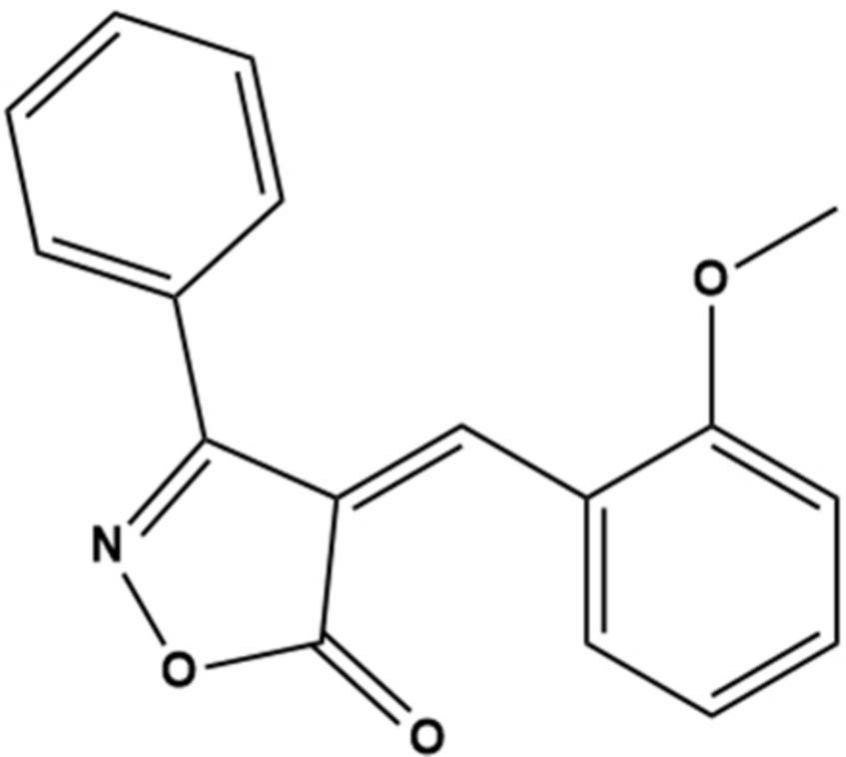


**Figure 1:**
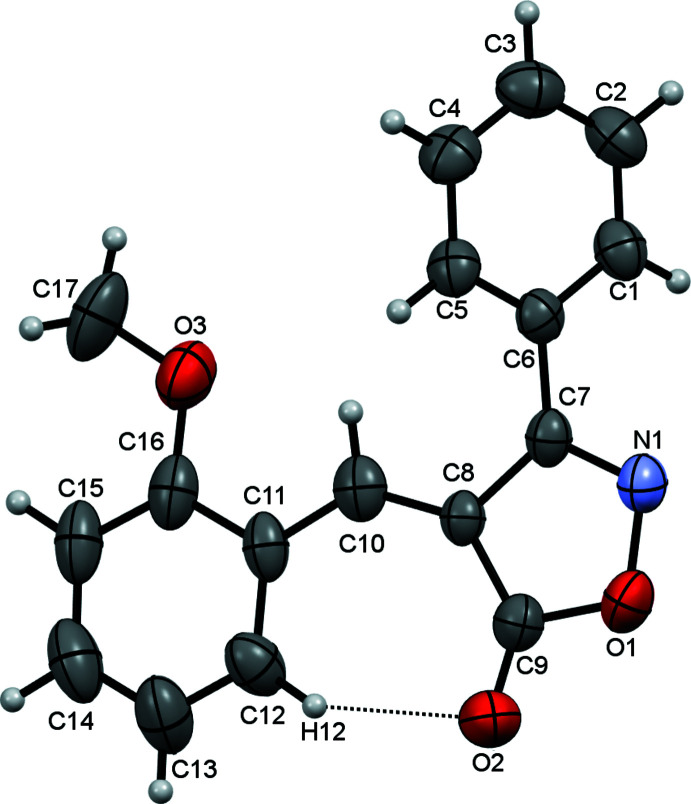
The mol­ecular structure of the title compound, with atom labelling and displacement ellipsoids drawn at the 50% probability level. The intra­molecular hydrogen bond is shown as a black dashed line.

We report here on the use of K_2_CO_3_ as very inexpensive, highly available and safe catalyst in an organic medium for isoxazolone formation and we describe the synthesis, mol­ecular and crystal structures, and Hirshfeld surface analysis of the title isoxazole derivative, **1** (Fig. 1 ▸).

## Structural commentary   

The asymmetric unit contains one mol­ecule and the mol­ecule adopts a *Z* configuration about the C8=C10 bond. The entire (*Z*)-4-(2-meth­oxy­benzyl­ideneisoxazolone) segment of the mol­ecule is almost planar with an r.m.s. deviation from the mean plane through all 15 non-hydrogen atoms of the fragment of only 0.0927 Å. This conformation is supported by the formation of an intra­molecular C12—H12⋯O1 hydrogen bond (Table 1 ▸), which links the isoxazole ring and the benzene ring of the 2-meth­oxy­benzyl­idene substituent. These two rings are inclined to one another at an angle of 9.63 (7)°. The (C1–C6) phenyl substituent is twisted out of this plane, the phenyl and isoxazole rings being inclined to one another by 46.22 (4)°. Bond lengths and angles agree well with those found in the isomeric derivative **2** (Zhang *et al.*, 2015 ▸) and also with the values observed for the related compound (4*Z*)-4-benzyl­idene-2-phenyl-1,3-oxazol5(4*H*)-one (Asiri *et al.*, 2012 ▸).

## Supra­molecular features   

**Figure 2:**
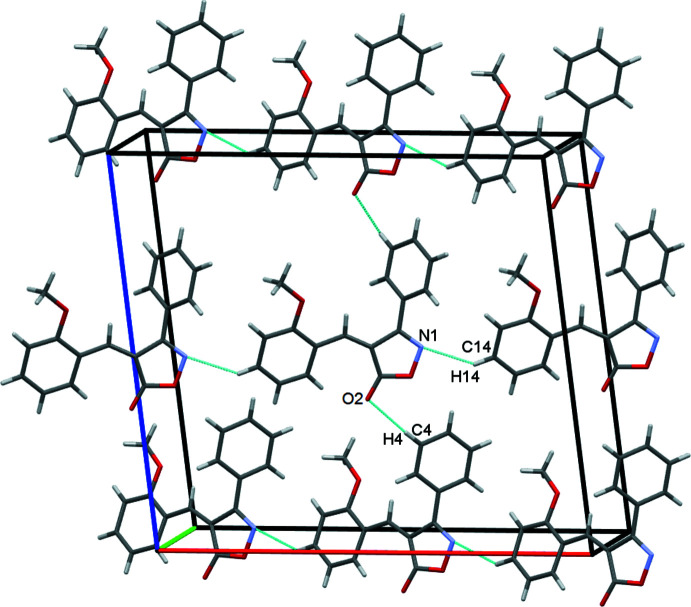
Sheets of mol­ecules of **1** in the *ac* plane.

**Figure 3:**
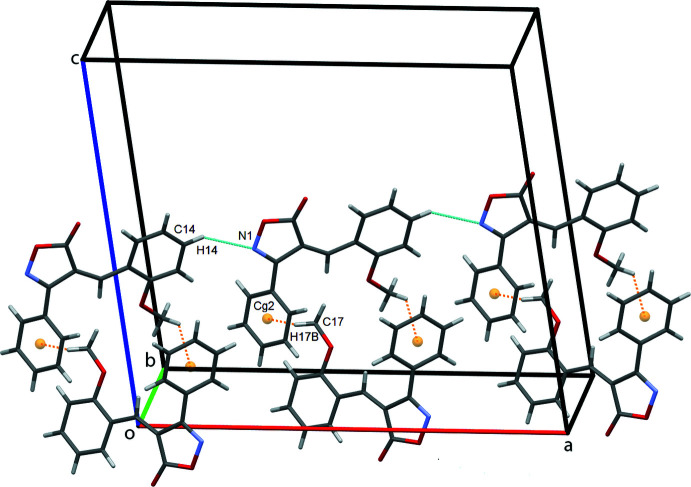
Double rows of mol­ecules of **1** along the *a-*axis direction. *Cg*2 is the centroid of the C1–C6 phenyl ring, shown here as an orange sphere, with the C—H⋯π contacts drawn as orange dashed lines.

**Figure 4:**
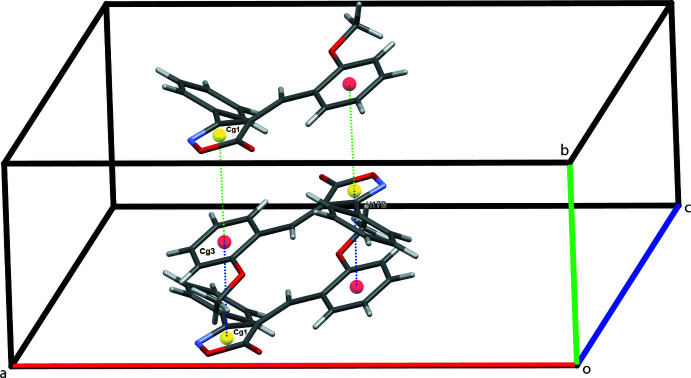
π–π contacts for 1 stacking mol­ecules along the *b*-axis direction. *Cg*1 and *Cg*3 are the centroids of the N1/O2/C7–C9 isoxazole and the C11–C16 benzene rings, respectively. The two discrete π–π contacts *Cg*1⋯*Cg*3 = 3.7049 (9) and 3.9200 (9) Å are shown as green and blue dashed lines, respectively.

In the crystal, mol­ecules stack along the *b*-axis direction (Fig. 2 ▸). Mol­ecules are connected by C4—H4⋯O1^i^ and C14—H14⋯N1^iii^ hydrogen bonds, leading to the formation of sheets in the *ac* plane, Fig. 3 ▸. C—H⋯π contacts between the meth­oxy­methyl group and the C1–C6 phenyl ring form double chains of mol­ecules along the *a*-axis direction, supported by the above-mentioned C14—H14⋯N1^iii^ hydrogen bonds, Fig. 4 ▸. Inter­molecular H⋯O short contacts are also present [C17—H17*A*⋯O3^iv^ = 2.78 Å and C5—H5⋯O2^ii^ = 2.81 Å]. Two π–π contacts [3.7049 (9) and 3.9200 (9) Å] are found between the centroids of the isoxazolone ring and the meth­oxy-substituted benzene ring, which stack adjacent mol­ecules in an obverse fashion along *b*.

## Analysis of the Hirshfeld surfaces   

**Figure 5:**
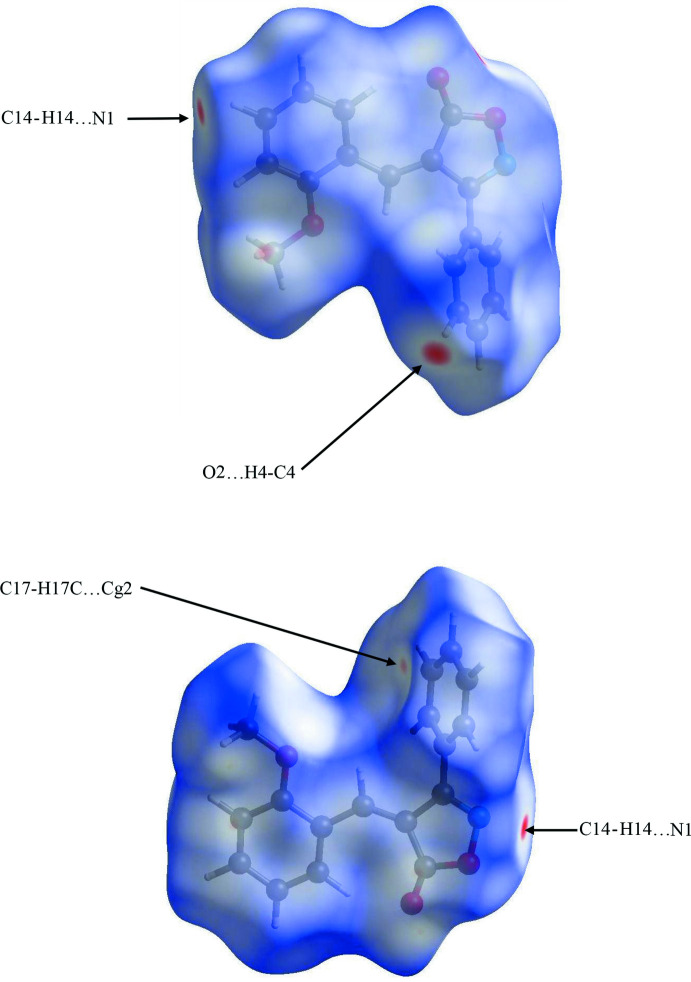
Hirshfeld surfaces for opposite faces of the mol­ecule of **1**, mapped over *d*_norm_ in the range −0.1701 to 1.4088 a.u.

**Figure 6:**
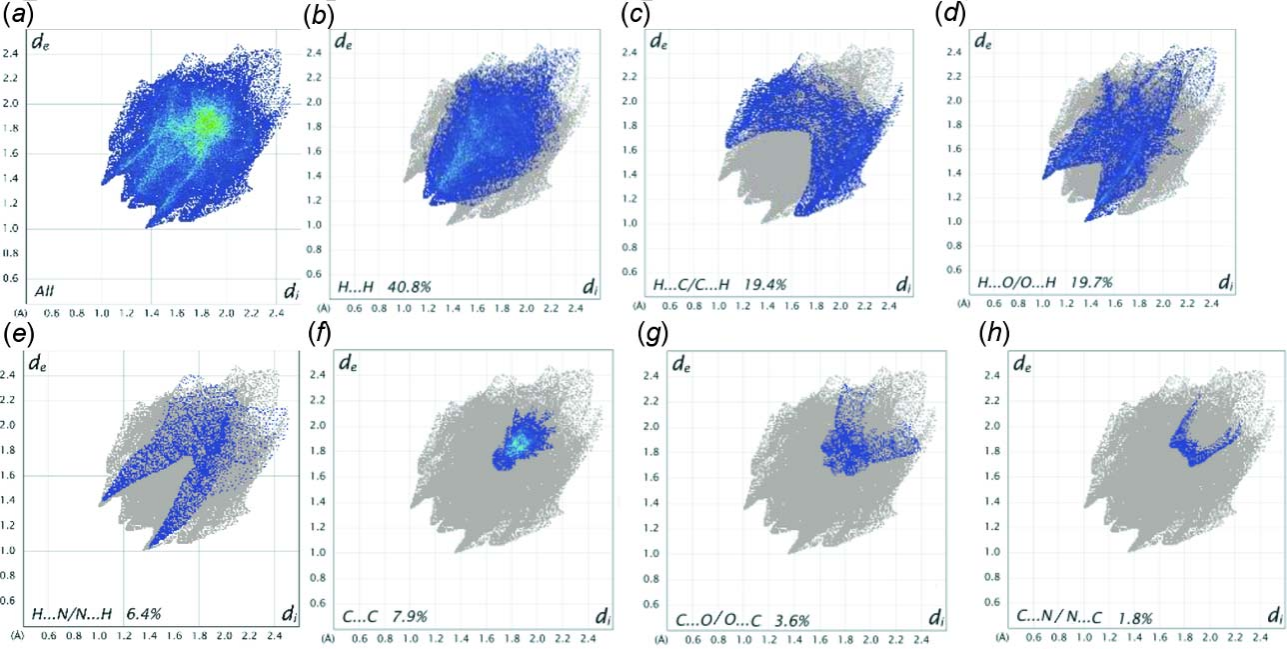
(*a*) The two-dimensional fingerprint plot for all inter­actions, together with those (*b*)–(*h*) delineated into individual contact types with included surface areas for the major individual contacts. Minor contacts contributing less than 1% to the total surface area are not shown here but, for completeness, are included in Table 2 ▸.

Further details of the inter­mol­ecular inter­actions in **1** were obtained using Hirshfeld surface analysis (Spackman & Jayatilaka, 2009 ▸) with Hirshfeld surfaces and two-dimensional fingerprint plots (McKinnon *et al.*, 2007 ▸) generated using *CrystalExplorer* (Turner *et al.* 2017 ▸). Fig. 5 ▸ shows the Hirshfeld surfaces for opposite faces of the asymmetric unit of mol­ecule **1**. The bright red circles correspond to C—H⋯N and C—H⋯O hydrogen bonds while a weaker C—H⋯π contact appears as a faint red circle. Fingerprint plots for **1** are shown in Fig. 6 ▸. As the CIF file for the isomeric mol­ecule, **2**, was available from the CCD, it was of inter­est to compare and contrast contributions to the included surface areas from the two isomers as shown in Table 2 ▸. As expected, H⋯H contacts are the most prolific in both cases. Other contributions were generally very similar, the sole exception being that the C⋯O/O⋯C contacts made up almost twice the surface area for **2** as for **1**. The change from the 2- to the 4-position in **2** may allow the meth­oxy substituent in **2** to contribute more substanti­ally to the surface of the mol­ecule.

## Database survey   

A search of the Cambridge Structural Database (CSD, V3.59, last update February 2019; Groom *et al.*, 2016 ▸) for (*Z*)-4-benzyl­idene-3-phenyl­soxazol-5(4*H*)-one yielded seventeen hits. Importantly, one of these, *i.e.* (*Z*)-4-(4-meth­oxy­benzyl­idene)-3-phenyl­isoxazol-5(4*H*)-one (SULZAC; Zhang *et al.*, 2015 ▸) is an isomer (**2**) of the title compound with the meth­oxy substituent in the 4-position of the benzene ring. Another paper (Jiang *et al.*, 2013 ▸) included the closely related compound (*Z*)-4-(4-[di­methyl­amino)benzyl­idene]-3-phenyl­isoxazol-5(4*H*)-one (IDIBEE) together with two other related compounds, IDIBII and IDIBOO, that exhibit large second harmonic generation effects. The search also revealed four other structures in which the configuration about the C=C bond is *Z*, namely 4-(2-hy­droxy­benzyl­idene)-3-methyl­isoxazol-5(4*H*)-one (AJESAK; Cheng *et al.*, 2009 ▸), (4*Z*)-4-benzyl­idene-2-phenyl-1,3-oxazol-5(4*H*)-one (YAXMUH; Asiri *et al.*, 2012 ▸), (*Z*)-4-benzyl­idene-3-methyl­isoxazol-5(4*H*)-one [MBYIOZ (Meunier-Piret *et al.*, 1972 ▸) and MBYIOZ01 (Chandra *et al.*, 2012 ▸)] and a recent addition, (*Z*)-4-(4-hy­droxy­benzyl­idene)-3-methyl­isoxazol-5(4*H*)-one (Zemamouche *et al.*, 2018 ▸).

## Synthesis and crystallization   

2-Meth­oxy­benzaldehyde (1 mmol), hy­droxy­amine hydro­chloride (1 mmol), ethyl benzoyl­acetate (1 mmol) and K_2_CO_3_ (5 mol%) were mixed in a 25 ml flask equipped with a magnetic stirrer. The mixture was refluxed in 5 ml of water for 2.5 h (the reaction was monitored by TLC). On completion of the reaction, the mixture was gradually poured into ice-cold water. Stirring was maintained for a few minutes and the resulting solid was filtered and purified by crystallization from ethanol.

## Refinement details   

Crystal data, data collection and structure refinement details are summarized in Table 3 ▸. H atoms were positioned geometrically (C—H = 0.93–0.96 Å) and refined as riding with *U*_iso_(H) = 1.2*U*_eq_(C) or 1.5*U*_eq_(C-meth­yl).

## Supplementary Material

Crystal structure: contains datablock(s) I. DOI: 10.1107/S2056989021004308/ex2043sup1.cif

Structure factors: contains datablock(s) I. DOI: 10.1107/S2056989021004308/ex2043Isup2.hkl

Supporting information file. DOI: 10.1107/S2056989021004308/ex2043Isup3.cml

CCDC reference: 2079211

Additional supporting information:  crystallographic information; 3D view; checkCIF report

## Figures and Tables

**Table 1:** Hydrogen-bond geometry (Å, °) *Cg* is the centroid of the C1–C6 ring.

*D*—H⋯*A*	*D*—H	H⋯*A*	*D*⋯*A*	*D*—H⋯*A*
C4—H4⋯O2^i^	0.93	2.53	3.463 (2)	176
C5—H5⋯O2^ii^	0.93	2.81	3.728 (2)	169
C10—H10⋯O3	0.93	2.26	2.7009 (18)	108
C12—H12⋯O2	0.93	2.15	2.998 (2)	151
C14—H14⋯N1^iii^	0.93	2.58	3.396 (2)	147
C17—H17*A*⋯O3^iv^	0.96	2.78	3.615 (2)	147
C17—H17*C*⋯*Cg* ^iv^	0.96	2.82	3.606 (2)	139

Symmetry codes: (i) x, -y+1, z+{\script{1\over 2}}; (ii) -x+1, -y+1, -z+1; (iii) x-{\script{1\over 2}}, y-{\script{1\over 2}}, z; (iv) -x+1, y, -z+{\script{3\over 2}}.

**Table 2:** Short contacts and contributions (%) to the Hirshfeld surface for **1** and **2**

Contact	**1**	**2**
H⋯H	40.8	40.5
H⋯C/C⋯H	19.4	18.1
H⋯O/O⋯H	19.7	19.6
H⋯N/N⋯H	6.4	5.3
C⋯C	7.9	6.5
C⋯O/O⋯C	3.6	6.9
C⋯N/N⋯C	1.8	2.9
O⋯O	0.6	0.1
N⋯N	0.1	

**Table 3:** Experimental details

Crystal data	
Chemical formula	C_17_H_13_NO_3_
*M*_r_	279.28
Crystal system, space group	Monoclinic, *C*2/*c*
Temperature (K)	293
*a*, *b*, *c* (Å)	20.3883 (6), 7.5925 (2), 17.9858 (5)
β (°)	95.791 (1)
*V* (Å^3^)	2769.96 (13)
*Z*	8
Radiation type	Mo *K*α
μ (mm^−1^)	0.09
Crystal size (mm)	0.32 × 0.23 × 0.10

Data collection	
Diffractometer	Bruker APEXII CCD
Absorption correction	Multi-scan (*SADABS*; Bruker, 2009 ▸)
*T*_min_, *T*_max_	0.98, 0.99
No. of measured, independent and observed [*I* > 2σ(*I*)] reflections	35544, 2495, 1548
*R*_int_	0.12
(sin θ/λ)_max_ (Å^−1^)	0.600

Refinement	
*R*[*F* ^2^ > 2σ(*F* ^2^)], *wR*(*F* ^2^), *S*	0.057, 0.101, 1.00
No. of reflections	2495
No. of parameters	190
H-atom treatment	H-atom parameters constrained
Δρ_max_, Δρ_min_ (e Å^−3^)	0.09, −0.16

Computer programs: *APEX2* and *SAINT* (Bruker, 2009 ▸), *SIR92* (Altomare *et al.*, 1994 ▸), *SHELXL2018/3* (Sheldrick, 2015 ▸), *ORTEP-3 for Windows* (Farrugia, 2012 ▸) and *Mercury* (Macrae *et al.*, 2020 ▸) and *WinGX* publication routines (Farrugia, 2012 ▸).

## supplementary crystallographic information

### Crystal data

**Table:**

C_17_H_13_NO_3_	*F*(000) = 1168
*M**_r_* = 279.28	*D*_x_ = 1.339 Mg m^−^^3^
Monoclinic, *C*2/*c*	Mo *K*α radiation, λ = 0.71073 Å
*a* = 20.3883 (6) Å	Cell parameters from 1218 reflections
*b* = 7.5925 (2) Å	θ = 2.3–33.4°
*c* = 17.9858 (5) Å	µ = 0.09 mm^−^^1^
β = 95.791 (1)°	*T* = 293 K
*V* = 2769.96 (13) Å^3^	Needle, white
*Z* = 8	0.32 × 0.23 × 0.10 mm

### Data collection

**Table:**

Bruker APEXII CCD diffractometer	1548 reflections with *I* > 2σ(*I*)
φ and ω scans	*R*_int_ = 0.12
Absorption correction: multi-scan (SADABS; Bruker, 2009)	θ_max_ = 25.3°, θ_min_ = 2.3°
*T*_min_ = 0.98, *T*_max_ = 0.99	*h* = −24→24
35544 measured reflections	*k* = −9→9
2495 independent reflections	*l* = −21→20

### Refinement

**Table:**

Refinement on *F*^2^	Primary atom site location: structure-invariant direct methods
Least-squares matrix: full	Secondary atom site location: structure-invariant direct methods
*R*[*F*^2^ > 2σ(*F*^2^)] = 0.057	Hydrogen site location: inferred from neighbouring sites
*wR*(*F*^2^) = 0.101	H-atom parameters constrained
*S* = 1.00	*w* = 1/[σ^2^(*F*_o_^2^) + (0.0548*P*)^2^] where *P* = (*F*_o_^2^ + 2*F*_c_^2^)/3
2495 reflections	(Δ/σ)_max_ < 0.001
190 parameters	Δρ_max_ = 0.09 e Å^−^^3^
0 restraints	Δρ_min_ = −0.16 e Å^−^^3^

### Special details

**Table:**

Geometry. All esds (except the esd in the dihedral angle between two l.s. planes) are estimated using the full covariance matrix. The cell esds are taken into account individually in the estimation of esds in distances, angles and torsion angles; correlations between esds in cell parameters are only used when they are defined by crystal symmetry. An approximate (isotropic) treatment of cell esds is used for estimating esds involving l.s. planes.

### Fractional atomic coordinates and isotropic or equivalent isotropic displacement parameters (Å^2^)

**Table:**

	*x*	*y*	*z*	*U*_iso_*/*U*_eq_	
O1	0.63879 (5)	0.40128 (15)	0.44786 (5)	0.0604 (3)	
O2	0.53785 (6)	0.34750 (15)	0.39356 (6)	0.0641 (4)	
O3	0.44474 (6)	0.07225 (16)	0.65984 (6)	0.0672 (4)	
N1	0.67268 (6)	0.40557 (18)	0.52191 (7)	0.0559 (4)	
C11	0.45217 (7)	0.17739 (18)	0.53778 (8)	0.0423 (4)	
C10	0.51760 (7)	0.23684 (17)	0.56343 (8)	0.0409 (4)	
H10	0.528401	0.219863	0.614403	0.049*	
C6	0.65205 (7)	0.35594 (18)	0.64799 (8)	0.0419 (4)	
C5	0.61267 (8)	0.43223 (19)	0.69787 (8)	0.0499 (4)	
H5	0.571966	0.479668	0.680373	0.060*	
C8	0.56690 (7)	0.31231 (18)	0.52928 (7)	0.0376 (4)	
C7	0.63081 (7)	0.35667 (18)	0.56711 (8)	0.0400 (4)	
C12	0.42284 (8)	0.1993 (2)	0.46438 (9)	0.0566 (5)	
H12	0.446785	0.253003	0.429297	0.068*	
C1	0.71264 (8)	0.2846 (2)	0.67501 (9)	0.0518 (4)	
H1	0.739346	0.232764	0.642170	0.062*	
C16	0.41449 (8)	0.0947 (2)	0.58918 (9)	0.0511 (4)	
C3	0.69408 (9)	0.3668 (2)	0.79957 (9)	0.0633 (5)	
H3	0.708286	0.370488	0.850331	0.076*	
C9	0.57442 (8)	0.3509 (2)	0.45076 (8)	0.0474 (4)	
C2	0.73315 (9)	0.2905 (2)	0.75030 (10)	0.0632 (5)	
H2	0.773723	0.242656	0.768033	0.076*	
C4	0.63365 (9)	0.4380 (2)	0.77336 (9)	0.0593 (5)	
H4	0.607189	0.489740	0.806480	0.071*	
C15	0.35023 (8)	0.0404 (2)	0.56730 (11)	0.0633 (5)	
H15	0.325381	−0.013537	0.601461	0.076*	
C14	0.32368 (9)	0.0672 (2)	0.49456 (12)	0.0717 (6)	
H14	0.280483	0.032695	0.480253	0.086*	
C13	0.35978 (9)	0.1436 (2)	0.44307 (11)	0.0710 (6)	
H13	0.341624	0.157705	0.393888	0.085*	
C17	0.41045 (9)	−0.0215 (3)	0.71332 (9)	0.0764 (6)	
H17A	0.437527	−0.026963	0.760129	0.115*	
H17B	0.370029	0.038399	0.720143	0.115*	
H17C	0.400829	−0.138807	0.695475	0.115*	

### Atomic displacement parameters (Å^2^)

**Table:**

	*U*^11^	*U*^22^	*U*^33^	*U*^12^	*U*^13^	*U*^23^
O1	0.0531 (8)	0.0810 (9)	0.0494 (6)	−0.0098 (6)	0.0165 (5)	0.0092 (5)
O2	0.0606 (8)	0.0864 (9)	0.0452 (6)	−0.0005 (6)	0.0045 (5)	0.0073 (5)
O3	0.0630 (8)	0.0842 (9)	0.0568 (7)	−0.0219 (6)	0.0176 (6)	0.0039 (6)
N1	0.0455 (9)	0.0667 (9)	0.0564 (8)	−0.0096 (7)	0.0101 (7)	0.0045 (7)
C11	0.0345 (9)	0.0357 (8)	0.0577 (9)	−0.0008 (7)	0.0086 (7)	−0.0027 (7)
C10	0.0416 (9)	0.0378 (9)	0.0437 (7)	0.0012 (7)	0.0055 (7)	−0.0010 (6)
C6	0.0362 (9)	0.0402 (9)	0.0494 (8)	−0.0053 (7)	0.0054 (7)	−0.0032 (7)
C5	0.0447 (10)	0.0503 (10)	0.0545 (9)	−0.0012 (8)	0.0036 (8)	−0.0042 (7)
C8	0.0354 (9)	0.0362 (8)	0.0421 (7)	−0.0012 (7)	0.0083 (6)	0.0011 (6)
C7	0.0353 (9)	0.0371 (9)	0.0490 (8)	−0.0024 (7)	0.0103 (7)	0.0008 (6)
C12	0.0477 (11)	0.0553 (11)	0.0653 (10)	−0.0036 (8)	−0.0009 (8)	0.0075 (8)
C1	0.0401 (10)	0.0558 (10)	0.0593 (9)	−0.0014 (8)	0.0042 (8)	−0.0076 (8)
C16	0.0454 (11)	0.0462 (10)	0.0639 (10)	−0.0059 (8)	0.0161 (8)	−0.0066 (8)
C3	0.0719 (14)	0.0657 (12)	0.0499 (9)	−0.0098 (10)	−0.0056 (9)	−0.0071 (8)
C9	0.0471 (11)	0.0485 (10)	0.0478 (8)	−0.0001 (8)	0.0109 (8)	0.0042 (7)
C2	0.0525 (11)	0.0653 (12)	0.0680 (11)	−0.0018 (9)	−0.0123 (10)	−0.0009 (9)
C4	0.0619 (13)	0.0622 (12)	0.0547 (9)	−0.0024 (9)	0.0112 (9)	−0.0136 (8)
C15	0.0442 (12)	0.0619 (12)	0.0865 (13)	−0.0126 (9)	0.0204 (9)	−0.0081 (10)
C14	0.0387 (11)	0.0725 (13)	0.1023 (15)	−0.0101 (9)	−0.0008 (11)	−0.0090 (11)
C13	0.0491 (12)	0.0754 (14)	0.0847 (13)	−0.0095 (10)	−0.0118 (10)	0.0106 (10)
C17	0.0838 (14)	0.0802 (14)	0.0719 (11)	−0.0161 (11)	0.0400 (10)	0.0042 (10)

### Geometric parameters (Å, º)

**Table:**

O1—C9	1.3729 (18)	C12—C13	1.371 (2)
O1—N1	1.4376 (15)	C12—H12	0.9300
O2—C9	1.2085 (17)	C1—C2	1.377 (2)
O3—C16	1.3663 (18)	C1—H1	0.9300
O3—C17	1.4341 (18)	C16—C15	1.392 (2)
N1—C7	1.2914 (18)	C3—C2	1.378 (2)
C11—C12	1.403 (2)	C3—C4	1.384 (2)
C11—C16	1.408 (2)	C3—H3	0.9300
C11—C10	1.4394 (19)	C2—H2	0.9300
C10—C8	1.3566 (19)	C4—H4	0.9300
C10—H10	0.9300	C15—C14	1.379 (2)
C6—C5	1.389 (2)	C15—H15	0.9300
C6—C1	1.391 (2)	C14—C13	1.369 (2)
C6—C7	1.4754 (19)	C14—H14	0.9300
C5—C4	1.3824 (19)	C13—H13	0.9300
C5—H5	0.9300	C17—H17A	0.9600
C8—C7	1.4475 (19)	C17—H17B	0.9600
C8—C9	1.4654 (18)	C17—H17C	0.9600
			
C9—O1—N1	110.05 (10)	C15—C16—C11	120.45 (16)
C16—O3—C17	118.75 (13)	C2—C3—C4	119.86 (15)
C7—N1—O1	106.87 (12)	C2—C3—H3	120.1
C12—C11—C16	117.50 (15)	C4—C3—H3	120.1
C12—C11—C10	123.89 (14)	O2—C9—O1	118.92 (13)
C16—C11—C10	118.61 (14)	O2—C9—C8	134.47 (15)
C8—C10—C11	133.82 (13)	O1—C9—C8	106.61 (12)
C8—C10—H10	113.1	C1—C2—C3	120.56 (16)
C11—C10—H10	113.1	C1—C2—H2	119.7
C5—C6—C1	119.18 (14)	C3—C2—H2	119.7
C5—C6—C7	120.26 (14)	C5—C4—C3	119.91 (16)
C1—C6—C7	120.52 (14)	C5—C4—H4	120.0
C4—C5—C6	120.39 (15)	C3—C4—H4	120.0
C4—C5—H5	119.8	C14—C15—C16	119.51 (17)
C6—C5—H5	119.8	C14—C15—H15	120.2
C10—C8—C7	123.95 (12)	C16—C15—H15	120.2
C10—C8—C9	132.47 (13)	C13—C14—C15	121.19 (17)
C7—C8—C9	103.27 (12)	C13—C14—H14	119.4
N1—C7—C8	113.07 (13)	C15—C14—H14	119.4
N1—C7—C6	118.35 (13)	C14—C13—C12	119.65 (17)
C8—C7—C6	128.56 (13)	C14—C13—H13	120.2
C13—C12—C11	121.68 (17)	C12—C13—H13	120.2
C13—C12—H12	119.2	O3—C17—H17A	109.5
C11—C12—H12	119.2	O3—C17—H17B	109.5
C2—C1—C6	120.10 (16)	H17A—C17—H17B	109.5
C2—C1—H1	119.9	O3—C17—H17C	109.5
C6—C1—H1	119.9	H17A—C17—H17C	109.5
O3—C16—C15	123.35 (16)	H17B—C17—H17C	109.5
O3—C16—C11	116.20 (14)		
			
C9—O1—N1—C7	1.14 (16)	C17—O3—C16—C15	4.0 (2)
C12—C11—C10—C8	−4.4 (3)	C17—O3—C16—C11	−175.92 (14)
C16—C11—C10—C8	176.65 (15)	C12—C11—C16—O3	178.84 (14)
C1—C6—C5—C4	0.4 (2)	C10—C11—C16—O3	−2.2 (2)
C7—C6—C5—C4	−177.39 (13)	C12—C11—C16—C15	−1.1 (2)
C11—C10—C8—C7	−177.90 (14)	C10—C11—C16—C15	177.91 (14)
C11—C10—C8—C9	−5.5 (3)	N1—O1—C9—O2	176.89 (13)
O1—N1—C7—C8	1.18 (17)	N1—O1—C9—C8	−2.88 (15)
O1—N1—C7—C6	−177.58 (11)	C10—C8—C9—O2	10.1 (3)
C10—C8—C7—N1	171.42 (14)	C7—C8—C9—O2	−176.37 (17)
C9—C8—C7—N1	−2.85 (17)	C10—C8—C9—O1	−170.22 (15)
C10—C8—C7—C6	−10.0 (2)	C7—C8—C9—O1	3.35 (15)
C9—C8—C7—C6	175.75 (14)	C6—C1—C2—C3	0.0 (2)
C5—C6—C7—N1	132.05 (16)	C4—C3—C2—C1	0.1 (2)
C1—C6—C7—N1	−45.7 (2)	C6—C5—C4—C3	−0.3 (2)
C5—C6—C7—C8	−46.5 (2)	C2—C3—C4—C5	0.1 (2)
C1—C6—C7—C8	135.77 (16)	O3—C16—C15—C14	−179.53 (15)
C16—C11—C12—C13	0.3 (2)	C11—C16—C15—C14	0.4 (2)
C10—C11—C12—C13	−178.59 (15)	C16—C15—C14—C13	1.1 (3)
C5—C6—C1—C2	−0.2 (2)	C15—C14—C13—C12	−1.9 (3)
C7—C6—C1—C2	177.52 (14)	C11—C12—C13—C14	1.1 (3)

### Hydrogen-bond geometry (Å, º)

*Cg* is the centroid of the C1–C6 ring.

**Table:**

*D*—H···*A*	*D*—H	H···*A*	*D*···*A*	*D*—H···*A*
C4—H4···O2^i^	0.93	2.53	3.463 (2)	176
C5—H5···O2^ii^	0.93	2.81	3.728 (2)	169
C10—H10···O3	0.93	2.26	2.7009 (18)	108
C12—H12···O2	0.93	2.15	2.998 (2)	151
C14—H14···N1^iii^	0.93	2.58	3.396 (2)	147
C17—H17*A*···O3^iv^	0.96	2.78	3.615 (2)	147
C17—H17*C*···*Cg*^iv^	0.96	2.82	3.606 (2)	139

Symmetry codes: (i) *x*, −*y*+1, *z*+1/2; (ii) −*x*+1, −*y*+1, −*z*+1; (iii) *x*−1/2, *y*−1/2, *z*; (iv) −*x*+1, *y*, −*z*+3/2.
